# Severe Fever with Thrombocytopenia Syndrome Virus, South Korea, 2013

**DOI:** 10.3201/eid2011.140888

**Published:** 2014-11

**Authors:** Sun-Whan Park, Myung-Guk Han, Seok-Min Yun, Chan Park, Won-Ja Lee, Jungsang Ryou

**Affiliations:** Korea Centers for Disease Control and Prevention, Cheongwon-gun, South Korea

**Keywords:** Severe fever with thrombocytopenia syndrome, Korea, Phlebovirus, Bunyaviridae, viruses

## Abstract

During 2013, severe fever with thrombocytopenia syndrome was diagnosed in 35 persons in South Korea. Environmental temperature probably affected the monthly and regional distribution of case-patients within the country. Phylogenetic analysis indicated that the isolates from Korea were closely related to isolates from China and Japan.

Severe fever with thrombocytopenia syndrome (SFTS) is a newly emerging infectious disease. Symptoms and laboratory abnormalities are fever, thrombocytopenia, leukocytopenia, and elevated serum enzyme levels. Multiorgan failure occurs in severe cases, and 6%–30% of case-patients die. The syndrome is caused by the SFTS virus (SFTSV) (genus *Phlebovirus*, family *Bunyaviridae*). SFTS case-patients were first reported in China (*1*) and more recently were reported in Japan (*2*) and South Korea (*3*). Two case-patients with symptoms consistent with a similar virus, Heartland virus, were reported in the United States (*4*).

Ixodid tick species are implicated as vectors of SFTSV (*1*,*5*,*6*). One study described a SFTSV prevalence in *Haemaphysalis longicornis* ticks, a major vector of SFTSV, of 0.46% minimum infection rate in South Korea (*7*); in another study, SFTSV was detected in ticks that had bitten humans (*6*). From these studies, we realized that SFTSV was common throughout the country. We aimed to evaluate the prevalence of SFTS in South Korea and isolate the SFTSV to analyze its phylogenetic properties.

## The Study

In March 2013, we established molecular diagnostic methods to detect SFTSV. During April–December 2013, from 125 hospitals throughout the country, we collected 301 serum samples from hospitalized persons who had SFTS signs and symptoms, such as high fever (temperatures >38°C), vomiting, diarrhea, and/or fatigue and showed laboratory parameters consistent with thrombocytopenia and/or leukocytopenia. We conducted reverse transcription PCR (RT-PCR) to detect the SFTSV medium (M) segment gene from acute-phase serum specimens with a previously described method (*6*). We also detected the SFTSV small (S) segment gene by RT-PCR with specific primers (SF3, 5′-GGGTCCCTGAAGGAGTTGTAAA-3′; SR1, 5′-TGGTGAGCAGCAGCTCAATT-3′). The RT-PCR conditions were as follows: an initial step of 30 min at 50°C for reverse transcription and 15 min at 95°C for denaturation, followed by 35 cycles of 20 s at 95°C, 40 s at 58°C (for M segment) or 55°C (for S segment), and 30 s at 72°C and a final extension step of 5 min at 72°C.

From the 301 samples, we detected M and S segment genes from 34 and 29 samples, respectively. The nucleotide sequences were assembled by the SeqMan program implemented in DNASTAR software (version 5.06; Madison, WI, USA) to determine the consensus sequences. The nucleotide sequences of the Korea isolates showed 93%–98% homology to the China and Japan isolates.

**Figure 1:**
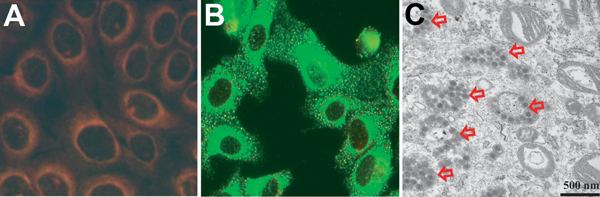
Isolation of severe fever with thrombocytopenia syndrome virus (SFTSV) from case-patients, South Korea, 2013. A, B) Indirect immunofluorescent features of Vero E6 cells primed with SFTSV N protein monoclonal antibody and reacted with fluoresce in isothiocyanateconjugated anti-mouse IgG. B) Transmission electron microscopy image of Vero E6 cells infected with SFTSV. Scale bar indicates 500 nm.

To isolate SFTSV, we inoculated subconfluent monolayers of Vero E6 cells with the RT-PCR–positive serum. After the monolayers underwent 3 blind passages in new monolayers of Vero E6 cells (*8*), we examined the Vero E6 cells for SFTSV by RT-PCR. We considered the virus to be isolated when the specific genes were amplified by RT-PCR. The viruses did not cause cytopathic effects in Vero E6 cells during isolation. Isolation of SFTSV also was confirmed by indirect immunofluorescent assay (IFA) (Figure 1, panels A,B) and electron microscopy (Figure 1, panel C). For IFA, Vero E6 cells infected with SFTSV were incubated at 37°C in a CO_2_ incubator. Cells were harvested, inoculated, and fixed with acetone on Teflon-coated well slides. IFA was conducted by using a monoclonal SFTSV nucleocapsid protein (N) antibody (manufactured in our laboratory) as the primary antibody. N proteins of SFTSV were distributed throughout the cytoplasm (Figure 1, panels A,B). By electron microscopy, Vero E6 cells infected with the SFTSV Korea isolate KAJJH showed bunyavirus-like particles, 80–100 nm in diameter, located in cytoplasmic vacuoles, presumably in the Golgi apparatus (Figure 1, panel C).

**Figure 2:**
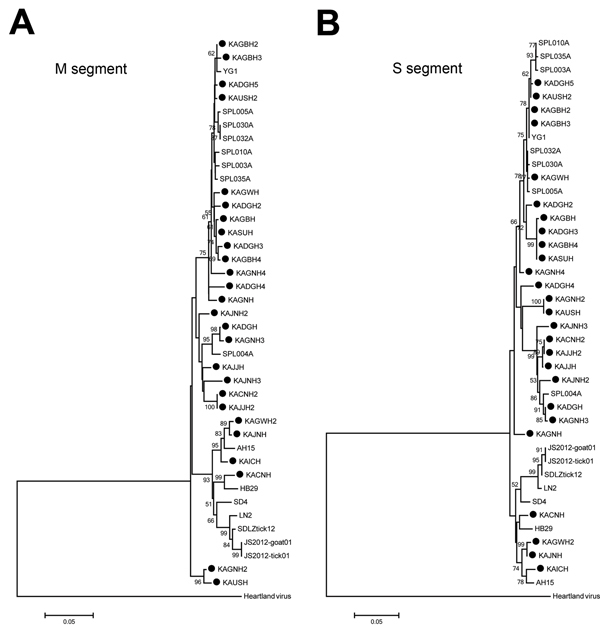
A and B) Phylogenetic analysis of SFTSV Korea isolates based on the partial medium (M) and small (S) segment sequences. The phylogenetic trees were generated by MEGA version 5.2 software (http://www.megasoftware.net/) from aligned nucleotide sequences of 16 isolates of phleboviruses, including the identified SFTSV. Heartland virus was used as outgroup. M and S partial nucleotide sequences of SFTSV Korea isolates were compared with homologous sequences of previously characterized SFTSVs. Sequences were analyzed by the neighbor-joining method based on the maximum composite likelihood model. The minimal length trees shown were supported as the majority rule consensus trees in 5,000 replicates. The bootstrap replicates supporting each node are indicated. Korea isolates are marked with black circles. Scale bar indicates nucleotide substitutions per site.

The amplified DNA products from the isolates were sequenced and compared with the sequences of other GenBank-registered SFTSV isolates. The sequences of partial M and S segments of the 26 Korea isolates (GenBank accession nos. KF282701, KF282702, and KJ739543–KJ739592) were closely related to those of the SFTSV isolates from China and Japan with 92%–100% identity. A phylogenetic tree was constructed by the neighbor-joining method on the basis of the partial M (Figure 2, panel A) and S segment (Figure 2, panel B) sequences of the Korea SFTSVs in the study and 15 SFTSVs from China and Japan registered in GenBank. SFTSV isolates formed 2 major clusters in M and S segment sequences, and 1 other small group comprising only Korea isolates, KAGNH2 and KAUSH, was formed in M-segment sequences. Many Korea isolates formed the first cluster with the Japan isolates. Some Korea isolates clustered with the major group of China isolates, forming the second group.

## Conclusions

We confirmed the SFTS in several localities around South Korea. We also isolated several SFTSVs from case-patient serum and analyzed the phylogenetic properties of the isolates. A total of 36 SFTS case-patients were reported in South Korea. The first SFTS case was identified in a retrospective study from 2012 (*3*). Subsequently, SFTS was diagnosed in 35 additional case-patients in South Korea. Another group diagnosed the first of the 35 cases in the country; we diagnosed the other 34 cases, from which we isolated the 26 SFTSVs. The major signs and symptoms of the 35 case-patients, including fever (100%), gastrointestinal symptoms (74%), fatigue (74%), thrombocytopenia (100%), and leukocytopenia (100%), were similar to those of case-patients in China and Japan (*9*).

**Figure 3:**
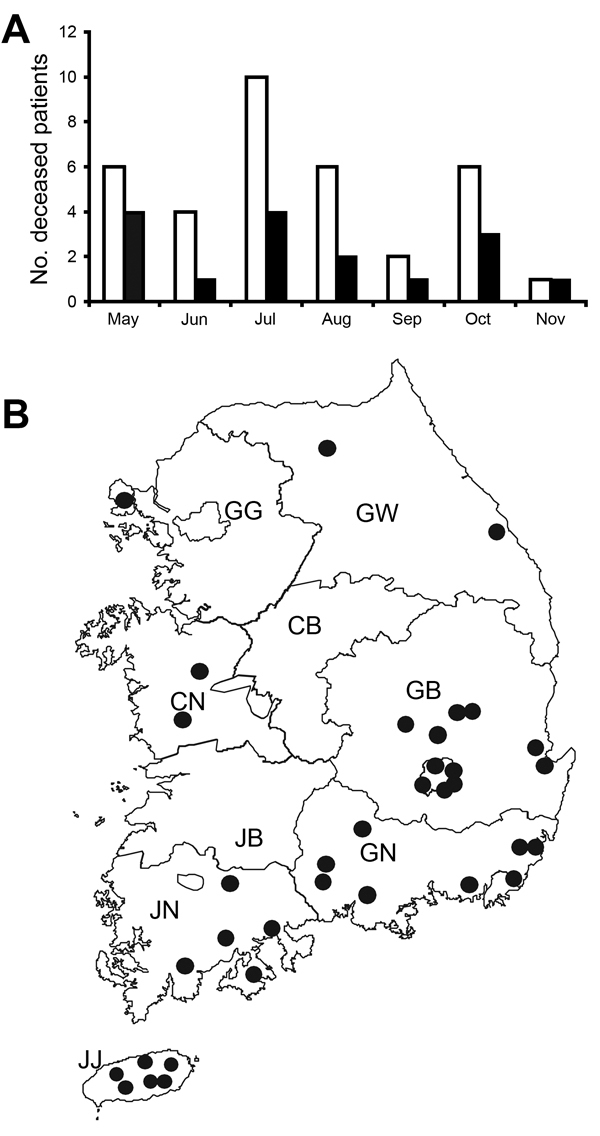
Seasonal (A) and geographic (B) distribution of casepatients with severe fever with thrombocytopenia syndrome (SFTS), South Korea, 2013. A) White and black bars indicate the numbers of total and deceased SFTS patients, respectively, in the indicated months. B) Black circles indicate the approximate residential regions of 35 SFTS case-patients in 2013 in South Korea. GG, Gyeonggi Province; GW, Gangwon Province; CB, Chungcheongbuk Province; CN, Chungcheongnam Province; GB, Gyeongsangbuk Province; GN, Gyeongsangnam Province; JB, Jeollabuk Province; JN, Jeollanam Province; JJ, Jeju special autonomous Province.

The case–fatality rate for SFTS in South Korea was 47.2% (17/36), higher than that of the recent China cases (≈8.7%) (*10*). The low sensitivity of the detection method, the conventional 1-step RT-PCR, and the absence of a serologic diagnosis may have contributed to the relatively high case–fatality rate. Most cases occurred in older persons; ≈80% of patientswere >50 years of age. Approximately 70% were farmers, including persons who cultivated vegetable gardens (*9*). In many case-patients, the disease evolved during a relatively warm time of year, from late spring to early autumn (Figure 3, panel A). The geographic distribution of SFTS case-patients also indicated that environmental temperature affected the SFTS prevalence because many (86%, 30/35) SFTS cases evolved in relatively warm southern provinces and cities south of Chungcheongbuk, Chungcheongnam, and Gangwon Provinces (Figure 3, panel B). We have also observed that the tick density is high during May–August, a generally warm season in South Korea (*7*). SFTSV was also mainly detected during this season. These results indicate that the virus infection in humans is closely related to a high tick density and SFTSV infection in ticks in a warm climate.

As described in another report, Japan isolates formed an independent cluster from the China isolates (*2*). In our current study, SFTSV isolates formed 2 major clusters. Most of the Korea isolates formed a cluster with the Japan isolates, although some Korea and China isolates were included in the other group, perhaps not surprising given the geographic location of South Korea between China and Japan.
